# Proton pump inhibitors use and risk of developing spontaneous bacterial peritonitis in cirrhotic patients: A systematic review and meta-analysis

**DOI:** 10.1186/s13099-021-00414-8

**Published:** 2021-03-19

**Authors:** Saad Alhumaid, Abbas Al Mutair, Zainab Al Alawi, Abdul Rehman Zia Zaidi, Ali A. Rabaan, Alyaa Elhazmi, Awad Al-Omari

**Affiliations:** 1 Administration of Pharmaceutical Care, Al-Ahsa Health Cluster, Ministry of Health, Al-Ahsa, Saudi Arabia; 2 Research Center, Almoosa Specialist Hospital, Al-Ahsa, Saudi Arabia; 3 College of Nursing, Princess Nourah Bint Abdul Rahman University, Riyadh, Saudi Arabia; 4 School of Nursing, University of Wollongong, Wollongong, Australia; 5 Department of Paediatrics, College of Medicine, King Faisal University, Al-Ahsa, Saudi Arabia; 6 Research Center, Dr. Sulaiman Al Habib Medical Group, Riyadh, Saudi Arabia; 7 College of Medicine, Alfaisal University, Riyadh, Saudi Arabia; 8 Molecular Diagnostic Laboratory, Johns Hopkins Aramco Healthcare, Dhahran, Saudi Arabia

**Keywords:** Ascites, Cirrhosis, Meta-analysis, Proton pump inhibitors, Spontaneous bacterial peritonitis, Systematic review

## Abstract

**Background:**

Spontaneous bacterial peritonitis (SBP) is one of the most common infectious diseases in patients with cirrhosis and is associated with serious prognosis. A prevailing dogma posits that SBP is exacerbated by the frequent use of proton pump inhibitors (PPIs).

**Aims:**

To re-assess the association between PPIs use and SBP incidence with larger and better-quality data.

**Method:**

The studies were identified by searching Proquest, Medline, and Embase for English language articles published between January 2008 and March 2020 using the following keywords alone or in combination: anti-ulcer agent, antacid, proton pump inhibitor, proton pumps, PPI, omeprazole, rabeprazole, lansoprazole, pantoprazole, esomeprazole, peritonitis, spontaneous bacterial peritonitis, SBP, ascites, cirrhosis, ascitic and cirrhotic. Three authors critically reviewed all of the studies retrieved and selected those judged to be the most relevant. Preferred

Reporting Items for Systematic Reviews and Meta-Analyses (PRISMA) statement was followed. Pooled odds ratios (ORs) with 95% confidence intervals (CIs) were calculated. Sub-group analyses were done to decrease the heterogeneity.

**Results:**

A total of twenty-three studies: seven case–control, and sixteen cohorts, involving 10,386 patients were analyzed. The overall results showed a statistically significant association between SBP and PPIs use (pooled odds ratio (OR): 1.80, 95% CI of 1.41 to 2.31). Substantial heterogeneity was observed. On subgroup analysis involving cohort studies, the association was weaker (OR: 1.55 with 95% CI of 1.16 to 2.06 *p* < 0.00001) but still statistically significant and with high heterogeneity (Chi^2^p = 57.68; *I*^*2*^ = 74%). For case–control studies, the OR was 2.62 with a 95% CI of 1.94 to 3.54. The funnel plot was asymmetric and Egger’s test confirmed asymmetry suggesting publication bias (intercept = − 0.05, SE = 0.27, P = 0.850 two-tailed).

**Conclusion:**

This meta-analysis sheds light on the conflicting results raised by previous studies regarding the association of SBP with PPIs use. Our meta-analysis showed that there is a weak association, although statistically significant, between SBP and PPIs use. However, the magnitude of the possible association diminished when analysis focused on higher quality data that were more robust. Thus, this updated meta-analysis suggests judicious use of PPIs among cirrhotic patients with ascites.

## Introduction

Spontaneous bacterial peritonitis (SBP) is defined as an ascitic fluid infection without an evident intra-abdominal surgically treatable source. Despite timely diagnosis and treatment its reported incidence in ascitic patients varies between 7 and 30% [1]. SBP should be suspected in a patient with ascites and any of the following: temperature greater than 37.8 °C (100°F), abdominal pain and/or tenderness, a change in mental status, or ascitic fluid polymorphonuclear leukocyte (PMN) count  ≥ 250 cells/mm^3^ [2]. SBP is one of the most common infectious diseases in patients with cirrhosis and is associated with a serious prognosis [3]. In-hospital mortality from SBP is estimated at 11–67% [4].

SBP is exacerbated by the frequent use of proton pump inhibitors (PPIs) in cirrhotic patients with ascites, leading to a reduction in gastric acidity and an increase in intestinal permeability which promotes bacterial translocation and colonization of mesenteric lymph nodes [5]. Subsequent infection of the fluid in the peritoneal cavity is also facilitated by the impairment of the body's defense mechanisms [6].

The use of PPIs has been widely reported to be associated with a higher incidence of SBP in hospitalized cirrhotic patients [7–11]. However, previous studies including case controls [7, 10], cohorts [8, 9, 11], and meta-analyses [12–14] provided conflicting conclusions. In light of newer studies that were done to re-evaluate the causality of PPI use and development of SBP, we aimed to re-assess the association between PPI use and SBP incidence with larger and better-quality data.

## Methods

### Design

We followed the Preferred Reporting Items for Systematic Reviews and Meta-Analyses guidelines (PRISMA) in conducting this systematic review and meta-analysis [16]. The following electronic databases were searched: PROQUEST, MEDLINE, and EMBASE with Full Text. Search keywords included anti-ulcer agents, antacids, proton pump inhibitors, proton pumps, PPI, omeprazole, rabeprazole, lansoprazole, pantoprazole, esomeprazole, peritonitis, spontaneous bacterial peritonitis, SBP, ascites, cirrhosis, ascitic and cirrhotic. The search was limited to papers published in English, between 1 January 2008 and 31 March 2020. The title and abstract of each selected article were read, and the article was retained if it discussed the use of PPIs and the development of SBP. A manual search through the bibliographies of the retrieved publications (backward snowballing) was conducted to increase the yield of potentially relevant articles.

### Inclusion–exclusion criteria

Articles were eligible for inclusion in this review and meta-analysis when they met all of these criteria: (1) observational study, including case control, and cohort study evaluating the risk of SBP associated with PPI therapy; (2) study population comprised adult patients (≥ 18 years); (3) SBP (defined as ≥ 250 polymorphonuclear leukocytes in the ascitic fluid) was a study endpoint; (4) hospital- or community-based study; and (5) date of publication between 2008 and 2020 in the English language. Articles were excluded if they met one of the following criteria: (1) editorials, commentaries, news analyses or reviews; (2) no control group of patients; (3) PPI therapy usage data (the type of therapy and who received the drug) was not available or could not be extracted, and (4) data were presented based on SBP episodes and not on the number of actual patients.

### Data extraction

Three authors (S.A., A.A. and Z.A.) critically reviewed all of the studies retrieved and selected those judged to be the most relevant. The abstracts of all citations were examined thoroughly. Data were extracted from the relevant research studies using key headings, which are noted in Table 1, simplifying analysis, and review of the literature. Articles were categorized as a case–control or a cohort study. The following data were extracted from selected studies: authors; publication year; study location; study design and setting; sample size, age, gender, and follow-up; statistical adjustment for confounders; and Newcastle–Ottawa Scale (NOS) score.

**Table 1:** General characteristics of included studies

Author, year [reference], study location	Case/control or case/cohort	Age (year), mean (SD)	Study design and setting	Male, n (%)	Adjusted for	Follow up (months)	NOS score
Aditi et al*.* 2012 [21]; USA	307/682	Not reported	Retrospective; cohort; single center	Not reported	Bilirubin, albumin, creatinine, INR, and protein in ascitic fluid	38 (mean)	7
Bajaj et al*.* 2009 [22]; USA	70/70	54.5 (13.0)	Retrospective; case–control; single center	79 (56.4)	CTP class, age, and admission time period	–	7
Campbell et al*.* 2008 [23]; USA	32/84	54.6 (10.7)	Retrospective; case–control; single center	78 (67.2)	Age, bilirubin, INR, creatinine, MELD score, DM, gender, history of SBP, etiology of liver disease, and race	–	5
Choi et al*.* 2011 [24]; Korea	83/93	55.5 (10.7)	Retrospective; case–control; single center	138 (78.4)	CTP class, MELD score, and VB	–	7
Cole et al*.* 2016 [25]; Scotland	114/92	20–74 (range)	Retrospective; cohort; single center	135 (65.5)	Age, MELD and UKELD scores, gender, etiology of liver disease, history of decompensate liver disease, and PPIs use	23.7 (median)	8
Dam et al*.* 2016 [26]; Denmark	340/525	57 (10.4)	Retrospective; cohort; multicenter	594 (68.8)	Use of PPIs; sex; age; cirrhosis etiology; VB; MELD score; increase of sodium, albumin, and platelets; dose of lactulose, spironolactone, furosemide and potassium-sparing diuretic	–	7
De Vos et al*.* 2013 [27]; Belgium	51/51	56 (8.9)	Retrospective; case–control; single center	70 (68.6)	None	1.5 (median)	7
Elzouki et al*.* 2019 [28]; Qatar	171/162	52.8 (12)	Retrospective; case–control; single center	260 (78.1)	Age, sex, DM, HTN, smoking, RF, and PPIs use	–	7
Goel et al*.* 2012 [7]; USA	65/65	57.6 (11.1)	Retrospective; case–control; single center	83 (63.8)	CTP classification	1 (mean)	8
Huang et al*.* 2016 [29]; Taiwan	1,870/1,190	54.1 (12.5)	Retrospective; cohort; multicenter	3,535 (73.8)	Age, sex, CAD, CHF, HTN, DM, CKD, ascites, HE, and esophageal varices	–	7
Janka et al*.* 2020 [30]; Hungary	74/39	50–64 (range)	Retrospective; cohort; Single center	69 (61.1)	Compensated stage, age, gender, comorbidity, etiology, MELD score and PPIs use	38.5 (median)	8
Khan et al*.* 2020 [31]; Pakistan	190/190	46.9 (10.1)	Prospective; cohort; Single center	220 (61.1)	Age, gender, etiology of liver disease, CTP score, albumin, bilirubin, and PT	–	6
Kim et al*.* 2017 [32]; Korea	58/249	57.7 (10.4)	Retrospective; cohort; single center	239 (77.8)	Age; sex; CTP score; SBP etiology; platelet count; ALT; GGT; BUN; creatinine; sodium; ascitic fluid protein; HC; H2RAs, PPIs, antibiotics, and Beta-blocker use	60 (mean)	7
Kwon et al*.* 2014 [33]; Korea	82/451	62.7 (9.5)	Retrospective; cohort; multicenter	410 (76.9)	Age, MELD score, H2RAs, and PPIs use	1 (mean)	7
Mandorfer et al*.* 2014 [8]; Austria	520/87	57.5 (11.8)	Retrospective; cohort; single center	426 (70.2)	Age, HC, history of variceal bleed, varices, and MELD score	9.6 (mean)	8
Min et al*.* 2014 [34]; Korea	402/402	57.7 (9.8)	Retrospective; cohort; single center	609 (75.7)	Age, gender, etiology of liver disease, CTP score, platelet count, GGT, BUN, creatinine, sodium, H2RAs, and PPIs use	25.1 (mean)	8
Miozzo et al*.* 2017 [35]; Brazil	151/107	54 (11.2)	Retrospective; cohort; single center	163 (63.4)	PPIs use, CTP and MELD scores, and the presence of upper gastrointestinal bleeding	60 (median)	6
Miura et al*.* 2014 [36]; Japan	18/47	66.3 (9)	Retrospective; cohort; single center	44 (67.7)	Age; gender; etiology of cirrhosis; DM; platelet count; creatinine; antibiotic, H2RAs and PPIs use; VB, HC, and HE; CTP and MELD scores and INR	–	5
O'Leary et al*.* 2015 [9]; USA	46/142	56.8 (9.3)	Prospective; cohort; multicenter	102 (54.1)	PPIs use, SBP prophylaxis, age, HR, MELD and CTP scores, platelet count, gender, sodium, albumin, MAP, index SBP infection, and number of infections	–	8
Rajender et al*.* 2019 [37]; India	143/143	51.5 (11.5)	Retrospective; cohort; single center	188 (65.7)	Age; gender; VB; HE; CTP and MELD scores; bilirubin; creatinine; cause of cirrhosis; ascitic fluid protein; PPIs, H2RAs and B blockers use	–	7
Ratelle et al*.* 2014 [10]; Canada	51/102	60.6 (15.1)	Retrospective; case–control; single center	114 (74.5)	PPIs use, gender, DM, sodium, and MELD score	–	8
Schiavon et al*.* 2017 [38]; Brazil	93/98	54.3 (12.5)	Prospective; cohort; Single center	130 (68.1)	Age, DM, previous hepatic encephalopathy and VB	32 (median)	7
Terg et al*.* 2015 [11]; Argentina	95/289	57.5 (11.5)	Retrospective; cohort; multicenter	265 (69)	Age, gender, MELD and CTP scores, alcohol, HE, bilirubin, albumin, creatinine, sodium, INR, platelet and leucocytes counts, and PPIs use	–	8

*AKI*: acute kidney injury, *ALT*: alanine aminotransferase, *AST*: aspartate transaminase, *BUN*: blood urea nitrogen, *CAD*: coronary artery disease, *CHE*: cholinesterase, *CHF*: congestive heart failure, *CKD*: chronic kidney disease, *CRP*: c-reactive protein, *CTP*: Child–Turcotte–Pugh class, *DM*: diabetes mellitus, *H2RAs:* H2 receptor antagonists, *HC*: hepatocellular carcinoma, *HE*: hepatic encephalopathy, *HIV*: human immunodeficiency virus, *HR*: heart rate, *HTN:* hypertension, *INR*: international normalized ratio, *GGT*: gamma-glutamyl transferase, *MAP*: mean arterial pressure, *MELD*: model for end-stage liver disease, *NOS:* Newcastle–Ottawa Scale, *PPIs*: proton pump inhibitors, *PT*: prothrombin time, *RF*: renal failure, *UKELD*: UK model for end-stage liver disease, *VB*: variceal bleeding

### Quality assessment

Newcastle–Ottawa Scale (NOS) was used to assess the quality of the selected studies [15]. This assessment scale has two different tools for evaluating case–control and cohort studies. Each tool measures quality in the three parameters of selection, comparability, and exposure/outcome, and allocates a maximum of 4, 2, and 3 points, respectively. High-quality studies are scored greater than 7 on this scale, and moderate-quality studies, between 5 and 7. Quality assessment was performed by two authors (SA and AA) independently, with any disagreement to be resolved by consensus.

### Data analysis

Meta-analyses were performed to calculate pooled odds ratios (ORs) with 95% confidence intervals (CIs). The similarity between the OR and other relative measures, such as RR, was assumed because SBP events and deaths were rare [17]. When both the crude and the adjusted OR/RR values were offered, only the adjusted value was adopted for the meta-analysis. If only the raw data was reported, we would calculate the unadjusted OR. Taking a conservative approach, a random effects model was used, which produces wider CIs than a fixed effect model.

Statistical heterogeneity was evaluated using the Cochran's chi-square (*χ*^*2*^) and the *I*^*2*^ statistic [18]. An *I*^*2*^ value of > 50% is suggestive of significant heterogeneity [19]. To detect the source of heterogeneity, subgroup analysis was performed based on study design (case–control or cohort), and quality of studies (high or moderate quality study). A sensitivity analysis was performed by excluding studies with relatively lower methodological quality. Publication bias was evaluated using funnel plots and the Egger’s correlation test, with P < 0.1 indicating statistical significance [20]. Review Manager (Version 5.3, Oxford, UK; The Cochrane Collaboration, 2014) and Stata (Version 13.0, Stata Corp, College Station, TX) were used to carry out all statistical analyses.

## Results

### Study characteristics and quality

**Fig. 1:**
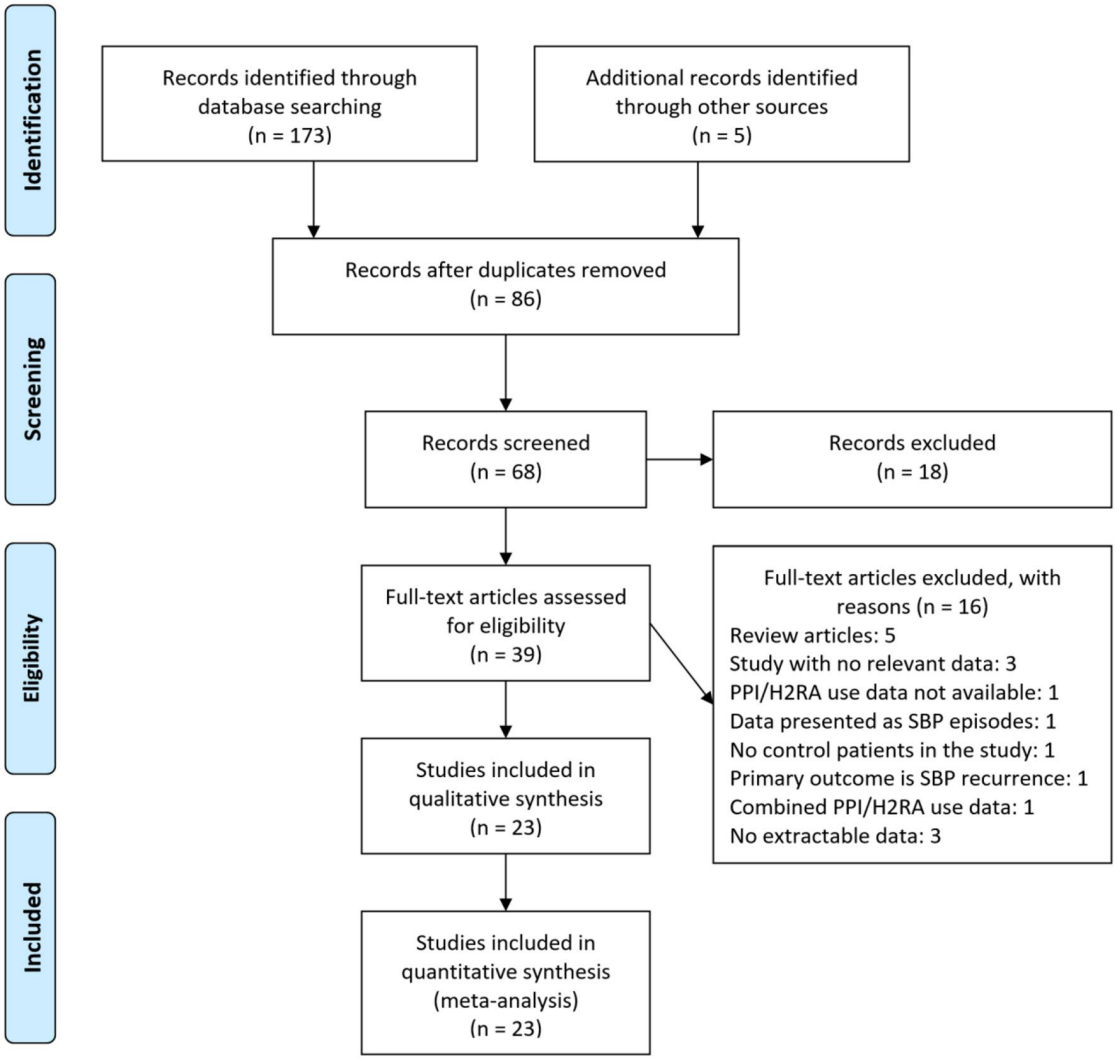
Flow diagram of studies included in the meta-analysis. *SBP* spontaneous bacterial peritonitis

A total of 178 publications were identified (Fig. 1). After scanning titles and abstracts, we discarded 86 duplicate articles. Another 18 irrelevant articles were excluded based on the titles and abstracts. The full texts of the 39 remaining articles were reviewed, and 16 irrelevant articles were excluded. As a result, we identified 23 studies that met our inclusion criteria [7–11, 21–38].

The detailed characteristics of the included studies are shown in Table 1. A total of 10,386 patients were included in the meta-analysis, 88.9% (9,236) of whom were part of cohort studies. There were 7 case–control studies and 16 cohort studies. These studies were conducted in North America, South America, Europe, and Asia. All studies adjusted the impact of confounders when assessing the association between PPIs use and SBP development except one study made by de Vos et al. [27]. The potential confounders most often adjusted for were age, Child–Turcotte–Pugh class, and Model for End-stage Liver Disease score. Only eight studies were performed with a multi-center design. The median NOS score for these studies was 7 (range, 5–8). Among the 23 included studies, 15 studies were moderate-quality studies (i.e., NOS scores were between 5 and 7) and 8 studies demonstrated a relatively high quality (i.e., NOS scores > 7; Table 1).

### Meta-analysis

**Fig. 2:**
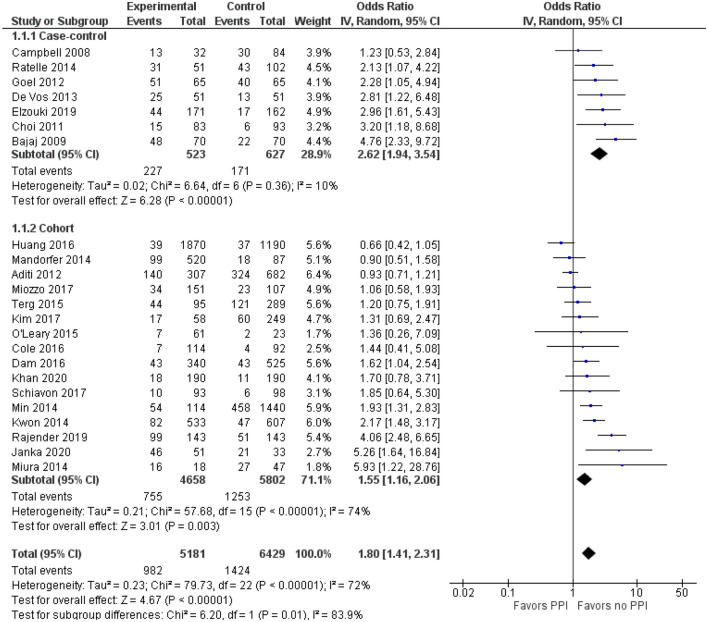
Forest plot for the association of SBP with PPIs use based on the design of the studies. *CI* confidence interval, *PPIs* proton pump inhibitors, *SBP* spontaneous bacterial peritonitis

The overall analysis of all 23 studies found that PPIs use was significantly associated with risk of SBP (OR = 1.80, 95% CI 1.41–2.31, *p* < 0.00001), with significant heterogeneity across studies (*I*^*2*^ = 72%, *p* < 0.00001). For the case–control studies, the pooled OR (95% CI) was 2.62 (1.94–3.54; *p* = 0.36; *I*^*2*^ = 10%). For the cohort studies, the pooled OR was 1.55 (95% CI 1.16–2.06, *p* < 0.00001; *I*^*2*^ = 74%; Fig. 2).

**Fig. 3:**
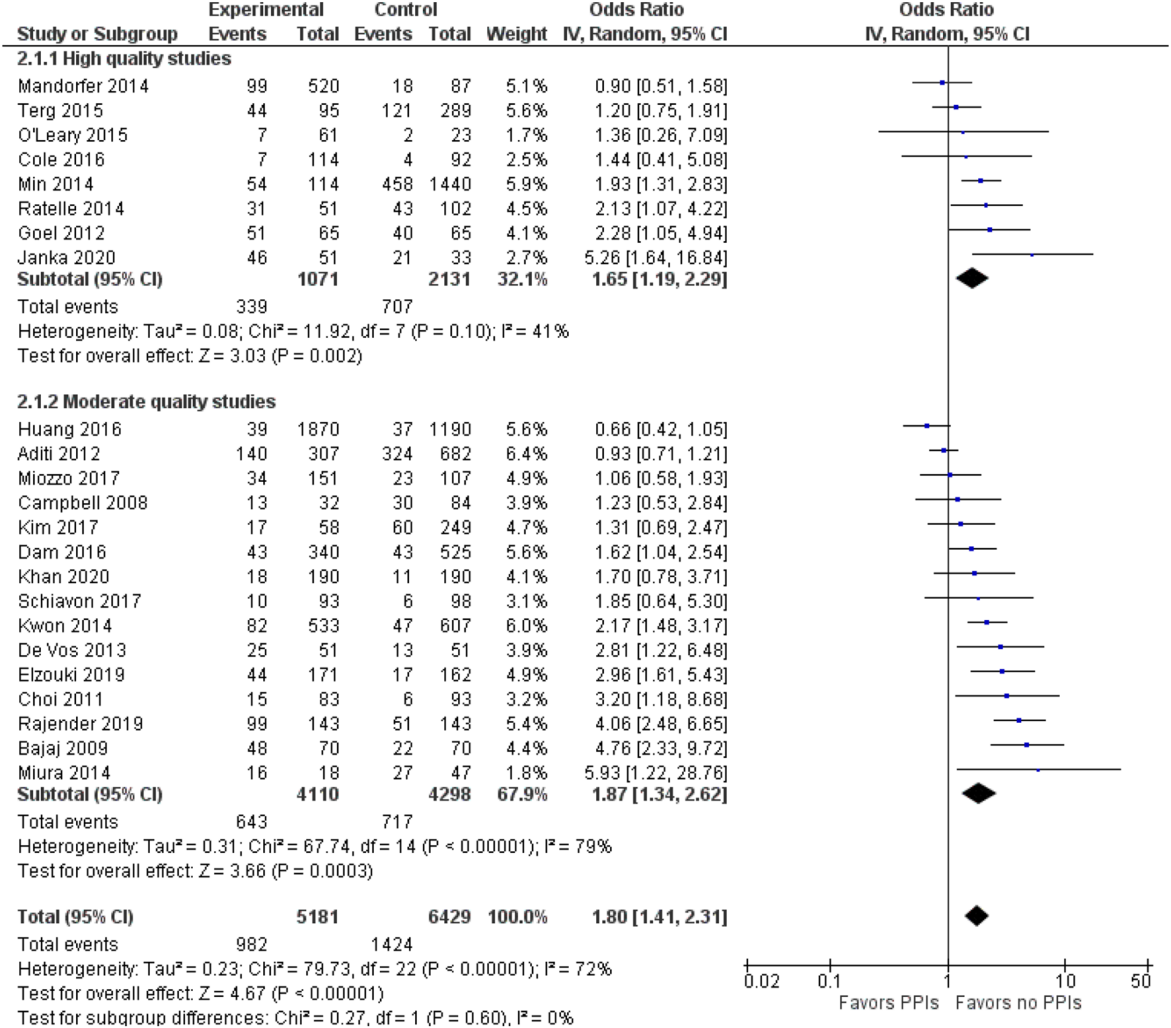
Forest plot for the association of SBP with PPIs use based on the quality of the studies. *CI* confidence interval, *PPIs* proton pump inhibitors, *SBP* spontaneous bacterial peritonitis

**Fig. 4:**
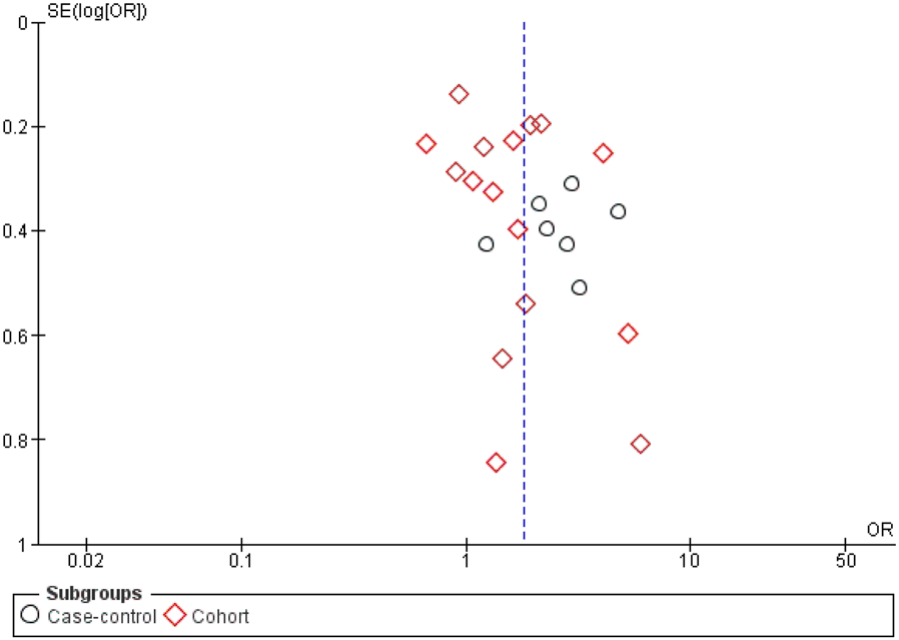
Funnel plot to evaluate publication bias (PPIs use and development of SBP). *OR* odds ratio, *PPI* proton pump inhibitor, *SBP* spontaneous bacterial peritonitis

Subgroup analysis was also carried out separately for high-quality and moderate-quality studies. The pooled OR for high-quality studies was 1.65 (95% CI 1.19–2.29, *p* = 0.10; *I*^*2*^ = 41%), and the pooled OR for moderate quality studies was 1.87 (95% CI 1.34–2.62, *p* < 0.00001; *I*^*2*^ = 79%; Fig. 3). The funnel plot for possible publication bias appeared asymmetrical on visual inspection, and Egger’s test confirmed asymmetry (intercept =—0.05, SE = 0.27, *p* = 0.850 two-tailed); Fig. 4.

## Discussion

This is the largest meta-analysis on the association between PPI use and risk of developing SBP in cirrhotic patients with/without ascites. This study involving 10,386 patients from 23 observational studies found statistically significant but quantitatively small associations between the development of SBP and the use of PPIs. The pooled data showed that PPIs use was associated with a 1.8-fold increased risk of developing SBP for cirrhotic patients. However, this harmful association was limited to cohort studies. The data from case–control studies demonstrated no causal relationships between the use of PPIs and SBP. The association was not statistically significant in the high-quality studies subgroup.

PPIs are used widely in clinical practice for a broad range of indications in patients. Indications for PPIs include the treatment of peptic ulcer disease, gastroesophageal reflux disease, Zollinger-Ellison syndrome, NSAID-associated ulcers, and eradication of *Helicobacter pylori* [39, 40]. They are also often used in patients with cirrhosis sometimes in the absence of a specific acid-related disease, with the aim of preventing peptic complications in patients with variceal or hypertensive gastropathic bleeding receiving multidrug treatment [41]. The use of this class of drugs seems more habit-related than evidence-based eventually leading to compromise patient safety and increase health costs [41]. Healthcare providers managing patients with cirrhosis should be aware of the fact that the use of PPIs is not justified in a majority of these patients and should make every effort to evaluate and reassess actively the existing PPI therapy. The use of PPIs by prescribers should be judicious and restricted for indications of proven benefit only.

Most studies involved in our systematic review showed that there was a risk between the use of PPIs and the development of SBP [7, 9, 10, 22, 24–26, 28–31, 33, 34, 36, 37], although few other included studies opposed this association [8, 21, 23, 27, 32, 35, 38]. The difference may be due to the patients with significant liver damage in the former fifteen studies. In addition, the mutant strains and its types, dosage of drugs may affect the results during treatment. The PPIs use and its association with the incidence of SBP in patients with cirrhosis is controversial, probably reflecting the heterogeneity of included patients across the studies and other methodological issues, such as retrospective design and insufficient follow-up. In addition, detrimental effects of PPIs may be restricted to specific subgroups, such as patients with decompensated cirrhosis, especially in the presence of ascites.

Although three latest previous systematic reviews have attempted to evaluate this association [12–14], our review is more current and more comprehensive. We included 21 published studies [7–11, 22–37] and 2 published abstracts [21, 38], with a higher patient population (n = 10,386); and the number of published studies in our analysis exceeds that in previous reviews. The inclusion of four studies published recently [28, 30, 31, 37] to our meta-analysis made a more precise estimate of the pooled OR effect size to evaluate PPI use and its association with incidence of SBP in cirrhotic patients.

## Limitations

There are several limitations to our findings. First, the included studies are observational in nature and, therefore, have intrinsic shortcomings, including differences in populations and possible unidentified confounders. Although some of these studies have suggested an association between PPIs therapy and SBP, they cannot establish causality with certainty. Well-designed, multi-center trials are needed for this purpose. To date, there are no prospective clinical trials, randomizing cirrhotic patients with/without ascites to PPIs use or non-use, which could be difficult to justify on clinical, ethical, or economic bases. Second, adjustment for the duration of PPIs was not possible therapy because many of the included studies did not report on the relevant data. Both duration and dose of PPI treatment should be related to the risk for the outcome of interest to support a causal association. Last, the exclusion of studies published in languages other than English may have impacted the richness of the data included in this review.

## Conclusion

Our meta-analysis of observational studies found that PPI use was associated with an increased risk of SBP in patients with cirrhosis with/without ascites. However, the magnitude of the possible association diminished when analysis focused on higher quality data that were more robust. PPIs can be used in the treatment of various therapeutic indications; nevertheless, PPIs therapy should be administered with caution in cirrhotic patients. Future studies maybe need to clarify the relationship between the occurrence of SBP and the type and dose of PPI in cirrhotic patients.

## Data Availability

Data are available upon request, please contact author for data requests.

## Abbreviations

SBP: Spontaneous bacterial peritonitis
PPIs: Proton pump inhibitors
PRISMA: Preferred Reporting Items for systematic reviews and meta-Analyses
NOS: Newcastle–Ottawa scale
PMN: Polymorphonuclear leukocyte
